# Single Trial EEG Patterns for the Prediction of Individual Differences in Fluid Intelligence

**DOI:** 10.3389/fnhum.2016.00687

**Published:** 2017-01-20

**Authors:** Emad-ul-Haq Qazi, Muhammad Hussain, Hatim Aboalsamh, Aamir Saeed Malik, Hafeez Ullah Amin, Saeed Bamatraf

**Affiliations:** ^1^Visual Computing Lab, Department of Computer Science, College of Computer and Information Sciences, King Saud UniversityRiyadh, Saudi Arabia; ^2^Centre for Intelligent Signal and Imaging Research (CISIR), Department of Electrical and Electronic Engineering, Universiti Teknologi PETRONASSeri Iskandar, Malaysia

**Keywords:** electroencephalography (EEG), fluid intelligence, cognitive task, discrete wavelet transform (DWT), machine learning classifier

## Abstract

Assessing a person's intelligence level is required in many situations, such as career counseling and clinical applications. EEG evoked potentials in oddball task and fluid intelligence score are correlated because both reflect the cognitive processing and attention. A system for prediction of an individual's fluid intelligence level using single trial Electroencephalography (EEG) signals has been proposed. For this purpose, we employed 2D and 3D contents and 34 subjects each for 2D and 3D, which were divided into low-ability (LA) and high-ability (HA) groups using Raven's Advanced Progressive Matrices (RAPM) test. Using visual oddball cognitive task, neural activity of each group was measured and analyzed over three midline electrodes (Fz, Cz, and Pz). To predict whether an individual belongs to LA or HA group, features were extracted using wavelet decomposition of EEG signals recorded in visual oddball task and support vector machine (SVM) was used as a classifier. Two different types of Haar wavelet transform based features have been extracted from the band (0.3 to 30 Hz) of EEG signals. Statistical wavelet features and wavelet coefficient features from the frequency bands 0.0–1.875 Hz (delta low) and 1.875–3.75 Hz (delta high), resulted in the 100 and 98% prediction accuracies, respectively, both for 2D and 3D contents. The analysis of these frequency bands showed clear difference between LA and HA groups. Further, discriminative values of the features have been validated using statistical significance tests and inter-class and intra-class variation analysis. Also, statistical test showed that there was no effect of 2D and 3D content on the assessment of fluid intelligence level. Comparisons with state-of-the-art techniques showed the superiority of the proposed system.

## Introduction

Individual differences are of wide practical importance in educational psychology and provide an opportunity to investigate concepts of cognitive functions (Gray et al., 2003). Fluid intelligence or general fluid intelligence (gf) is a major measurement of individual differences, which reflects the ability of reasoning and solving novel problems, i.e., tasks that cannot be solved as a function of simple memorization. It is also indirectly related to learning, memory retention and recall process (Ricardo et al., 2010). Various studies on cognitive tasks have linked the fluid intelligence with human learning ability and capacity (Deary et al., 2007; Van den Bos et al., 2012; Wang et al., 2013), which cannot be assessed subjectively i.e., by mere memorization and answering the questions (Primi et al., 2010). The alternative is to use direct brain activations, for which EEG signals can be used.

Electroencephalography (EEG) is a commonly used neuroimaging technique to analyze neural processing and can be used to assess a subjects' fluid intelligence level. A common approach to study cognitive processes is to use event-related potentials (ERP), a component of EEG. Recent research has shown that ERP exhibited variations while subjects performed various cognitive tasks (Polich, 2007; Ubeyli, 2009; Wronka et al., 2013). ERP represents averaged time locked brain voltage variations in EEG recordings, which are related to cognitive tasks. Due to averaging, ERP can be misleading because it might not reflect the actual brain dynamics of subjects (Gaspar et al., 2011). On the other side, single trial EEG signals provide the information that is not accessible using the conventional analysis of peak amplitudes and latencies of ERP (Quiroga et al., 2007). Single-trial analysis can provide a systematic mapping between (i) brain activity and stimulus information space (Schyns, 2010; Rousselet et al., 2011), (ii) brain activity and subject's behavioral variability (Ratcliff et al., 2009), and (iii) brain activity measured using different imaging techniques, e.g., fMRI and EEG (Goldman et al., 2009; deBettencourt et al., 2011). The focus of most of the previous research was on the classification of EEG signals based on different cognitive tasks and rest condition tasks, i.e., eyes open and eyes closed (baseline tasks), and no one addressed the problem of assessing the intelligence level of individuals.

In order to assess a subject's fluid intelligence level, we employed single trial EEG signals and assumed two fluid intelligence levels, i.e., LA and HA. As such, it was modeled as a two-class classification problem. To model a classification system, we collected EEG signals from 34 subjects while watching 2D and 3D contents. One pattern recognition system was developed for 2D and 3D each. Firstly, the RAPM test was used to divide the subjects into two groups (i.e., LA and HA) based on their intellectual ability. Next, we used the visual oddball cognitive task to measure the neural activity of each group by presenting target and standard stimuli and measured the brain activation as EEG signals from three sites *Fz, Pz, Cz*. After preprocessing the EEG signals, Haar wavelet transform was used to extract the statistical wavelet features (SWF) and wavelet coefficient features (WCF) from the low-frequency bands. Then the state-of-the-art classification technique, i.e., SVM with RBF kernel was used for our system to predict whether a subject belongs to LA or HA group. The proposed system gives promising results for the prediction of fluid intelligence level.

The main contributions of this study are (i) a system for the prediction of a subject's fluid intelligence level using single trial EEG signals, (ii) two different feature descriptors based on Haar wavelet transform, which are easy to compute and are effective in discriminating LA and HA groups, (iii) the analysis of low frequency bands showing that there is a clear difference between EEG signals belonging to LA and HA groups. To validate the discriminative values of the features, we employed the statistical significance test, and inter-class and intra-class variation analysis.

The rest of the paper is organized as follows: In Section Literature Review we present the literature review. Section Materials and Methods describes in detail the proposed method. Experimental results and discussion are given in Section Experimental Results and Discussion, while Section Conclusion concludes the paper.

## Literature review

The prediction of a subject's fluid intelligence level is a classification problem, which involves extracting discriminatory features from EEG signals and classification. Though, according to our knowledge, no study have been conducted which deal with the prediction of a subject's fluid intelligence level so far, there have been a number of studies on the classification of EEG signals for various similar cognitive tasks. In the following paragraphs, we reviewed those methods, which used wavelet transform (WT), time and frequency-domain techniques for feature extraction (Iscan et al., 2011).

WT, time and frequency-domain techniques have been widely used to extract features from cognitive and rest conditions' tasks. Out of these techniques, WT showed very good performance as compared to other methods due to its compliance with the EEG brain signals which having non-stationary behavior. The WT features which are considered for analysis are statistical features (standard deviations, mean and median) (Yazdani et al., 2009; Garry et al., 2013), wavelet entropy (Rosso et al., 2001) and wavelet coefficients (Orhan et al., 2011). These features have been used for analysis in EEG and clinical applications. The features based on time domain are Lyapunov exponent (Ubeyli, 2010), Hurst component (Acharya et al., 2012), Hjorth parameters, fractal dimension, permutation entropy (Vidaurre et al., 2009), approximate entropy and sample entropy (Richman and Moorman, 2000). Frequency-domain features in various frequency bands are power ratio, EEG absolute power and relative power (Thatcher et al., 2005). The analysis of time–frequency signals include stockwell transform and WT based feature extraction (Hariharan et al., 2014). Guo et al. (2011) utilized the immune feature along with the weighted SVM. The author performed the classification of cognitive tasks and achieved the accuracy between 85.4 and 97.5%. Hariharan et al. (2014) utilized the stockwell transform for the extraction of discriminatory features. They carried out the classification by using the SVM. For this purpose, they used the EEG signals recorded from various cognitive tasks. Accuracy rate of 84.72 to 98.95 % was achieved by the authors during the classification phase. Zhang et al. (2010) used the Fischer's discriminant classifier and high-frequency power for the classification of EEG signals, recorded during the cognitive tasks. They achieved the classification accuracy between 72.4 and 76.4%. Hosni et al. (2007) used the power feature of EEG signals. For classification, they utilized the SVM with a radial basis function (RBF) kernel. They achieved the accuracy of 70% by performing the classification on three cognitive tasks.

Xue et al. (2003) achieved the accuracy of 85.3% by using the RBF classifier. They used the wavelet packet transform as a feature extractor. In another study, Zhiwei and Minfen (2007) used the wavelet pack entropy as a feature. By applying the SVM classifier, they achieved the accuracy between 87.5 and 93.0%. In this scheme, they showed the discrimination between cognitive and baseline task. In the experiment, five tasks were performed by the database of seven subjects, i.e., (1) eyes open (baseline) task, (2) mental letter composing task, (3) geometric object rotation task, (4) multiplication task and (5) visual counting task. Keirn and Aunon (1990) developed this database at Colorado State University, which was based on simple cognitive tasks consisting of seven subjects only. In some research studies, authors utilized the database of few subjects for the classification purpose. As in Zhiwei and Minfen (2007), authors utilized only the database of two subjects. Similarly, in another research study, Nai-Jen and Palaniappan (2004) used the database of four subjects in their experimentation. Researchers also worked on classification of EEG brain signals, which were recorded during different cognitive tasks, by utilizing different databases developed by authors in their research studies. Lin and Hsieh (2009) achieved the accuracy of 78.31% by using the neural network classifier. They used the EEG power features for the classification of cognitive tasks. Rodrıguez-Bermudez et al. (2013) achieved the accuracy of 67.96–80.71 % by utilizing the wavelet, time and frequency based features. They used the SVM as classifier. In another research study, Karkare et al. (2009) utilized the scaling exponent as a feature. Artificial neural network was utilized as a classifier to classify the two groups. These two groups did the cognitive task of complex nature. They achieved the accuracy of 80%. The above-discussed studies showed the low accuracy of classification. Many of these studies used the non-linear classifiers like artificial neural networks. These classifiers were time consuming in constructing the models for the classification purpose. Jahidin et al. (2014) used the progressive metric test, i.e., Raven as a cognitive task. They obtained the accuracy of 88.89%. They utilized the EEG power and artificial neural network as a feature and classifier, respectively.

The above literature review indicates that wavelet transform is the most effective tool for extracting discriminative features from EEG signals. As such, we felt motivated to employ WT to propose an efficient feature extraction and classification (online and offline) system that can predict the fluid intelligence level of the subjects, whether they belong to HA or LA group by performing the cognitive task using 2D and 3D contents.

## Materials and methods

The objective of this study is to develop a system for predicting the fluid intelligence level of a subject, whether he/she belongs to low or high ability group. One system is developed for 2D and 3D content each, for assessing the intelligence level.

**Figure 1:**
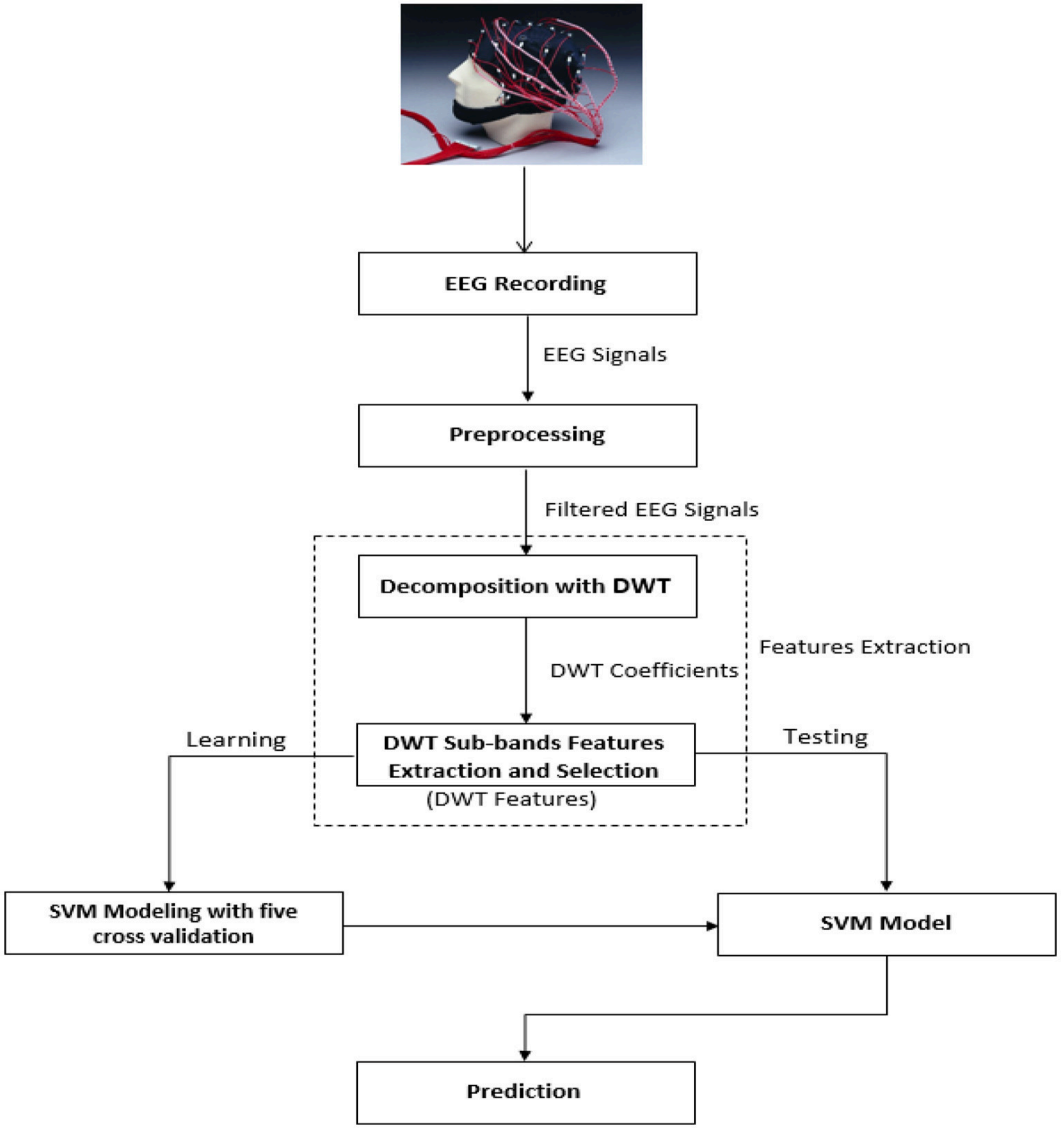
**Proposed methodology for feature extraction and classification of EEG signals**.

First, we selected the material, which consisted of 2D and 3D contents and the subjects for experiments to collect the data. The subjects were divided into LA and HA groups using RAPM test. In most of the applications, initially it is enough to identify weather a subject belongs to low or high ability group (Amin et al., 2015a). Next, EEG signals were recorded by performing the visual oddball cognitive task for neural activity. These EEG signals were preprocessed to remove the artifacts and noisy signals. Then discriminatory features were extracted from the preprocessed signals by using discrete wavelet transform. Finally, the most representative and relevant features were selected, which were input to the classifier to predict the intelligence level. An overview of the system is given in Figure 1.

### Experimental material and subjects

For performing experiments, to collect data for each of 2D and 3D contents, 34 healthy male subjects were selected to participate in cognitive tasks. They were all healthy students. 31 were right-handed, and the remaining three were left handed students. Their age range was from 20 to 30 years. They were all medically fit and free from neurological disorders and hearing impairments, and were not using any medication. They possessed corrected to normal or normal vision. All subjects were briefed about the experiment. All of them showed their consent and signed the consent form before the test. In visual oddball cognitive task, Target (sphere) and Standard (box) stimuli were shown to the subjects as 2D and 3D contents. The Human Research Ethics Committee of the Universiti Sains Malaysia and Ethics Coordination Committee of the Universiti Teknologi PETRONAS approved this research study.

### Data collection procedure

Before the start of the experiment, each subject was briefed about the procedure of data collection and the schedule. According to the availability of subjects, the experiment was carried out on the individual basis. Before the experiment, each subject was asked to solve 10 descriptive questions. These questions acted as a pre-test for the subjects. They were related to experimental learning content. The purpose of these questions was to control the subject's background knowledge. Ten percent was the exclusion criteria. It means that maximum one right answer was allowed from each subject. If he/she gave more than one correct answer, then he/she was excluded from the test. No subject indicated the past background information about the experimental learning contents used as a part of this analysis. It confirmed the balance between the LA and HA groups. Every subject was briefed on the experimental methodology, and their seating was arranged in partly sound attenuated room. Then all subjects were given the RAPM test to divide into two groups, i.e., LA and HA. The number of subjects in HA and LA groups in 2D case were 17 each, whereas, in 3D case, the number of subjects were 15 in HA group and 19 in LA group. Next, each subject participated in the visual oddball task. During this task, EEG cap was mounted on the subject's head to capture the EEG signals. The duration of the experiment was about 04 min. Each subject was seated about 1.5 meters away from the TV screen. The size of the screen, on which the task was shown, was about 41 inches. E-Prime Professional, version 2.0 (Psychology Software Tools, Inc., Sharpsburg, PA) was utilized for designing and implementation of this task (Schneider et al., 2002). The detail of the above-mentioned cognitive tasks has been discussed in the next sections.

### Cognitive tasks

#### Raven's advanced progressive matrices (RAPM) test

**Figure 2:**
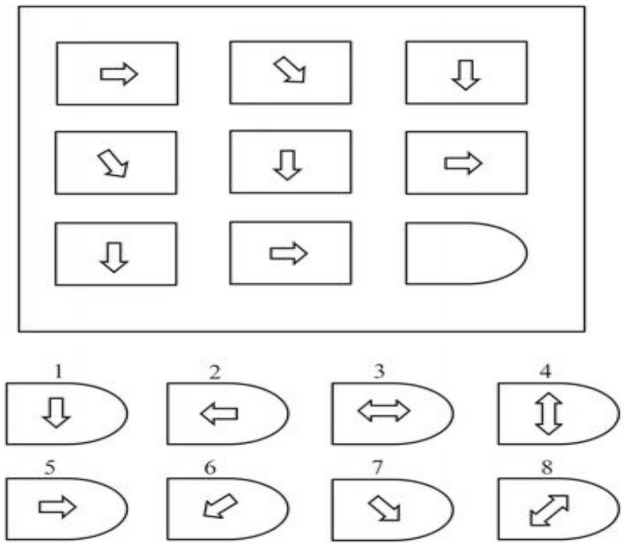
**An example of RAPM test (Amin et al., 2015a)**.

Raven's Advanced Progressive Matrices (RAPM) test (Raven, 2000) is a non-verbal test, which is used to measure the intelligence level of a subject. RAPM measures the two types of fluid cognitive ability, i.e., (i) ability to draw meaning out of confusion, and (ii) ability to recall and reproduce information that has been made explicit and communicated from one to another. It comprises 48 patterns, which are divided into two sets (I and II). Set-I contains 12 patterns, which are used for practice; Set-II contains 36 patterns that are used to assess cognitive ability. Each pattern consists of nine 3 × 3-cells, where each cell represents a geometrical shape except the right-bottom cell, which is empty and is to be filled from given eight options. A sample is shown in Figure 2. A subject has to fill the empty cells in 36 patterns, each carrying one score. A score of “1” is assigned for each correct answer and a score of “0” for an incorrect answer. Total scores range from 0 to 36. Processing time ranges from 10 min for Set-I to 40 min for Set-II (Raven, 2000; Amin et al., 2013). Details of RAPM scores of LA and HA groups for 2D and 3D cases are shown in Table 1.

**Table 1:** **Details of RAPM Scores of HA (High Ability) and LA (Low Ability) Groups for 2D and 3D cases**.

**No. of subjects**	**2D Case**				**3D Case**			
	**HA**		**LA**		**HA**		**LA**	
	**Subject ID**	**Score**	**Subject ID**	**Score**	**Subject ID**	**Score**	**Subject ID**	**Score**
1	1	27	2	21	1	26	2	22
2	3	24	4	17	3	24	4	17
3	8	32	5	13	5	33	8	21
4	9	29	6	21	6	28	13	23
5	10	28	7	17	7	24	14	23
6	11	28	12	22	9	27	15	19
7	17	27	13	19	10	32	16	16
8	18	29	14	21	11	34	19	23
9	19	31	15	12	12	32	20	13
10	22	30	16	18	17	24	21	17
11	24	25	20	20	18	24	22	22
12	26	29	21	22	26	26	23	23
13	27	24	23	13	30	30	24	21
14	28	26	25	23	31	25	25	15
15	32	24	29	6	34	25	27	14
16	33	28	30	19	-	-	28	23
17	34	31	31	21	-	-	29	19
18	-	-	-	-	-	-	32	23
19	-	-	-	-	-	-	33	20

*Median in 2D case = 23.5 and 3D case = 23*.

**Figure 3:**
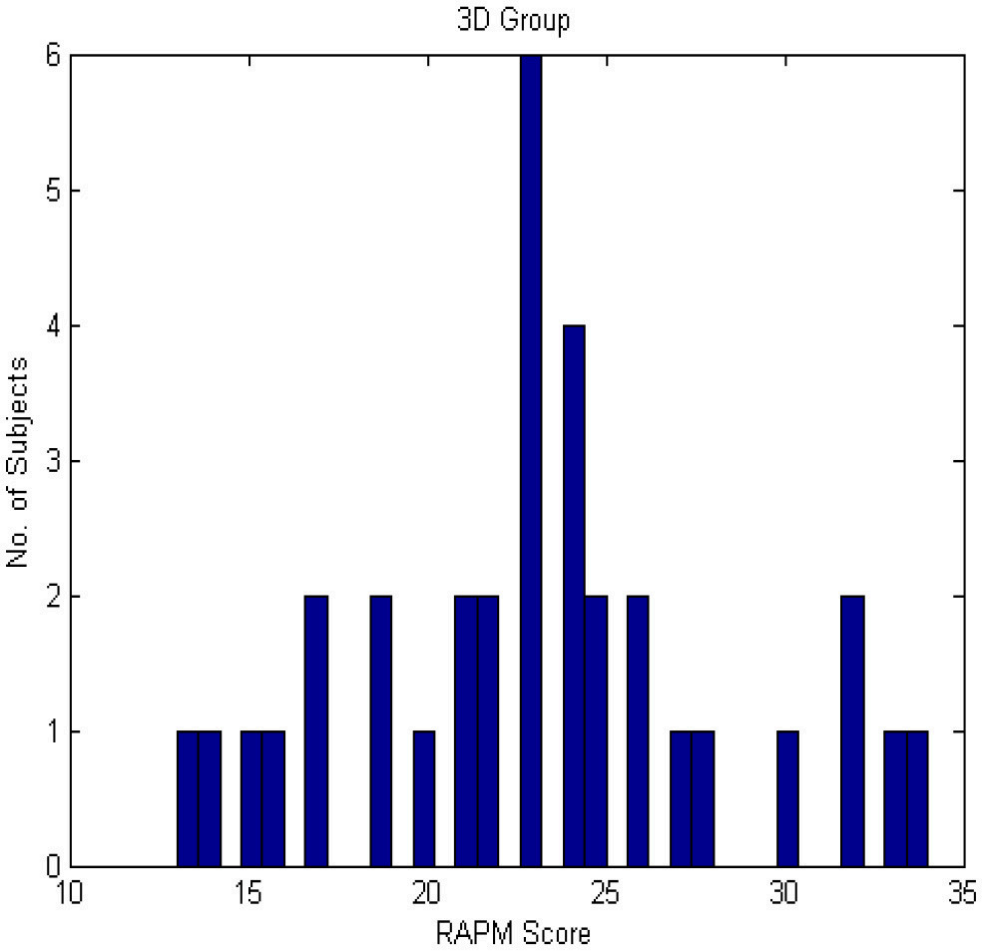
**Histogram of RAPM score for 3D group**.

**Figure 4:**
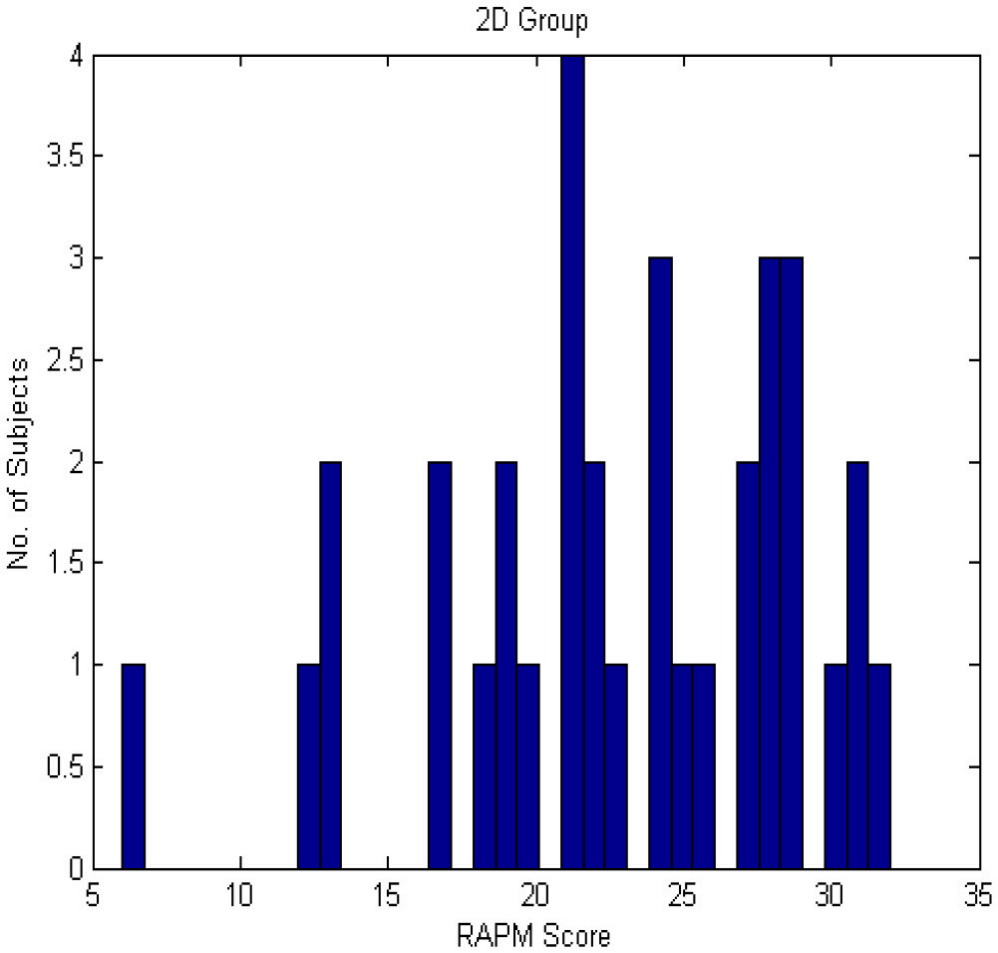
**Histogram of RAPM score for 2D group**.

RAPM scores for both the groups were roughly normally distributed as shown in Figures 3, 4.

No subject was eliminated because each subject's RAPM score was between MEAN − 3SD (22.85 − 18.48 = 4.37) and MEAN + 3SD (22.85 + 18.48 = 41.33) in 2D case. Similarly, in 3D case, each subject's RAPM score was between MEAN − 3SD (23.18 − 15.84 = 7.34) and MEAN + 3SD (23.18 + 15.84 = 39.02). Moreover, the mean age of the two groups was almost similar. In case of 2D, the mean age of HA group was 23.39 (*SD* = ± 3.29) years and that of LA group was 24.05 (*SD* = ± 2.25) years. In case of 3D, it was 22.81 (*SD* = ± 2.65) years for HA group and 24.24 (*SD* = ± 2.73) years for LA group.

#### Visual oddball task

The visual oddball task is commonly used for ERP research studies. In this study, visual stimuli were shown to subjects to invoke the neural activities in the attention and cognitive demanding events (Polich, 2007). All selected subjects participated in the visual oddball task. During visual oddball cognitive task, we used two types of stimuli (standard, which was box large, medium, or small box and target, which was a sphere). Both target and standard stimuli had the same size of 5 cm. Each stimulus appeared on a screen for a duration of 500 ms; there was a pre-stimulus period of 100 ms before the appearance of each stimulus. The subject was required to press “0” when a target shape appeared, and no response was required when standard shapes appeared. Subjects were instructed to respond as quickly as possible to avoid errors. Between the appearances of two stimuli, a black screen was displayed for 1000 ms. There were 30% target trials and 70% non-target (standard) trails. As such, out of 135 (total) trials, 40 were target trials, which were used for experiments. The task was performed in accordance with the modification recommended in Huettel and McCarthy (2004).

### EEG recording

**Figure 5:**
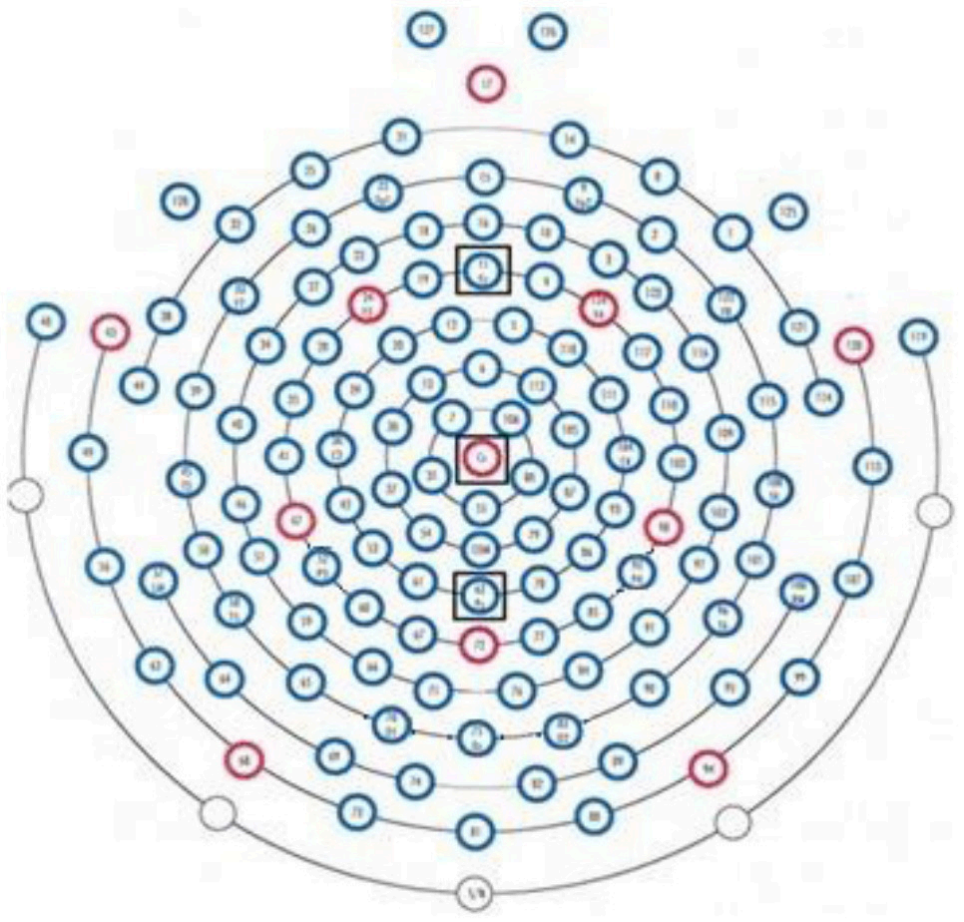
**Placement of Electrodes (HydroCel Geodesic Net; Amin et al., 2015a)**.

For recording EEG signals from each subject while undergoing visual oddball task, we used the HydroCel Geodesic Sensor Net (Electrical Geodesic Inc., Eugene, OR, USA). It consisted of 128 scalp electrodes as shown in Figure 5. The study by Jongsma et al. (2012) for tracking recall performance and event-related potentials (ERPs) across multiple trials in a digit-learning task used midline electrodes (*Fz, Cz, Pz*). In addition, while studying the (visual or cognitive fatigue) effects of stereoscopic 3D display technology on ERP components, Amin et al. (2015b) also used these midline electrodes. Motivated by these studies, we employed these electrodes to predict the fluid intelligence level of a subject. And our findings indicated that these electrodes are most suitable for this purpose. Therefore, we used only three midline channels to record the EEG signals, i.e., *Fz, Pz, Cz*. 10–20 International System was used for the placement of 128 electrodes on the scalp. The 250 Hz was used as a sampling rate and 50 KΩ was utilized as an upper limit of impedance. *Cz* was used as a single vertex electrode and all other electrodes were referenced from it. Amplification of raw signals was carried out from *Cz* with the EGI NetAmps 300 amplifier's band pass filter (0.1–100 Hz).

From previous studies, it is known that different brain regions may be associated to different brain functions. We used three channels (*Fz, Pz, Cz*) for EEG recording due to following reasons (Teplan, 2002):

- *Fz* near intentional and motivational centers,
- *Pz* contribute to activity of perception and differentiation,
- *Cz* location deals with sensory and motor functions.

### Data preprocessing

The recorded EEG signals involved artifacts. In order to remove the artifacts NetStation v4.5.4 software (Electrical Geodesic, Inc. Eugene, OR, USA) was used to preprocess the EEG signals. First, for removing the muscular artifacts of high frequency and DC components, a band pass filter was utilized (roll off 12 dB octave, 0.3–30 Hz). Next, segmentation of EEG trials related to each subject was performed by using a window of duration 600 mS, which contains the baseline, i.e., pre-stimulus period of 100 mS and post stimulus period of 500 mS. The trials which involved artifacts like eye movements and eye blinks were rejected, for example, if the amplitude of the EEG signal of any trial was ±90 μV then it was rejected. Visual inspection was used for all the trial segments and the contribution of electrodes, which had no contact in the phase of widespread drift (Balas and Koldewyn, 2013), was removed. Spherical spline method (Ferree, 2000) was used to discard a trial if any bad channel was found.

### Feature extraction

The single trials or epochs belong to two classes, i.e., LA and HA group. After preprocessing, discriminatory features are extracted from EEG signals of each trial. In this section, we proposed two techniques for feature extraction utilizing DWT, commonly used to analyze the biomedical signals based on their time-frequency content (Jahankhani et al., 2006; Orhan et al., 2011). An overview is given in the feature extraction module of Figure 1.

The band passed EEG signal (0.3–30 Hz) corresponding to one epoch consists of three components corresponding to the channels *Fz, Pz, and Cz*, we represent it as follows:

(1) $$\begin{array}{r} X\left(t\right)=\left[f\left(t\right),\;p\left(t\right),\;c\left(t\right)\right] \end{array}$$

where *f*(*t*), *p*(*t*) and *c*(*t*) correspond to *Fz, Pz* and *Cz* channels.

**Figure 6:**
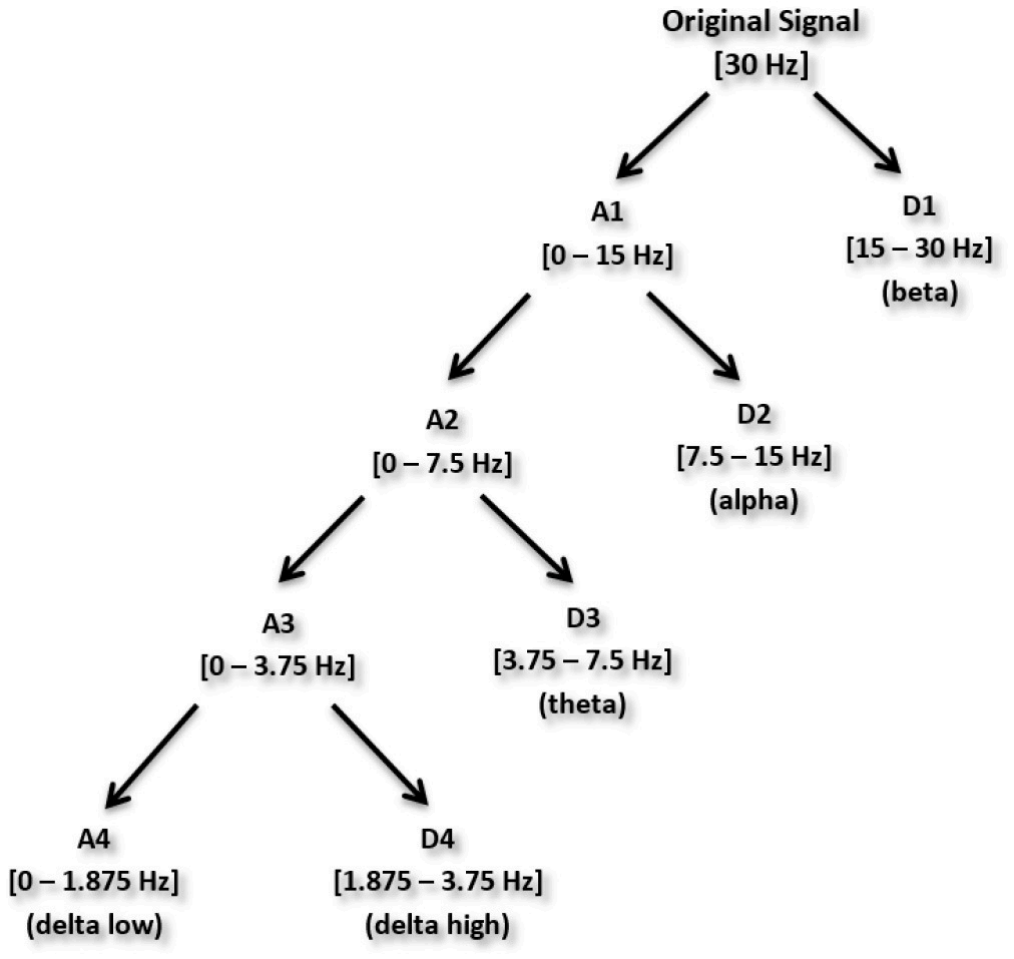
**Wavelet decomposition process up to level-4 (Jahankhani et al., 2006)**.

First, DWT with Haar wavelet was used to decompose each of *f*(*t*), *p*(*t*) and *c*(*t*) into sub-bands. This resulted in approximate and detail coefficients as shown in Figure 6.

#### Selection of wavelet decomposition level

The maximum number of decomposition levels depends on the required frequency components of the signals (Akay, 1997; Adeli et al., 2003; Subasi, 2007; Ocak, 2008). We decomposed a signal upto level 4.

**Figure 7:**
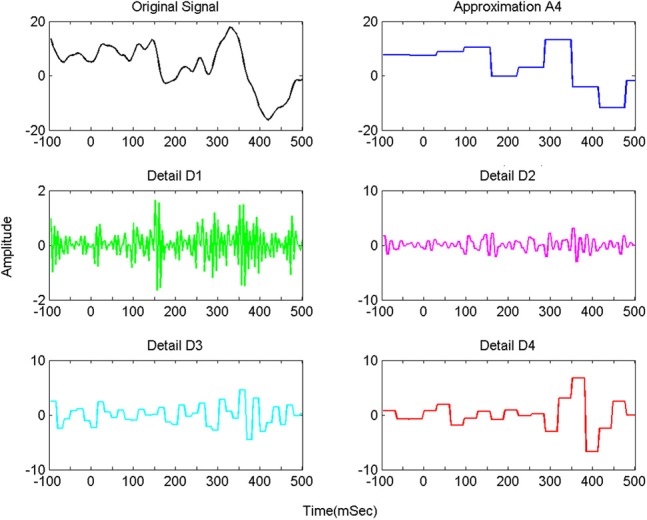
**Decomposition of single trial EEG signal of one subject at *Fz* channel**.

In previous studies (Dimitriadis et al., 2010; Harper et al., 2014), delta bands have been associated with attention and cognitive tasks. The studies involving the event-related potential (ERP) revealed the relationship of delta band with cognitive processes i.e., P300 component is associated with cognitive process (Ergen et al., 2008; Harper et al., 2014). Gennady (2012) reviewed the relationship of delta band with cognitive processes and confirmed this association. These studies reported significant increase in delta power during cognitive tasks. The range of delta band is from 0 to 3.75 Hz and the decomposition shown in Figure 6 indicates that $A_{4}^{s}\text{ and }D_{4}^{s}$ bands at decomposition level 4 represent delta band. As such, we decomposed the signal upto level 4. The $A_{4}^{s},D_{4}^{s},\;D_{3}^{s},\;D_{2}^{s}\text{ and }D_{1}^{s}$ components of a subject's EEG signal of single trial at *Fz* channel are shown in Figure 7.

The high-pass filter *g*(*n*) is the discrete mother wavelet, and the low-pass filter *h*(*n*) is its mirror version (Soltani, 2002; Orhan et al., 2011). The approximate and detail coefficients are computed by convolving each component of *X*(*t*) with the translates and dilates ϕ_*j, k*_(*t*), ψ_*j, k*_(*t*) of scaling function ϕ(*t*) and wavelet function ψ(*t*), respectively, defined as follows:

(2) $$\begin{array}{r} \phi_{j,k}\left(t\right)=2^{\frac{j}{2}}h\left(2^{j}n-k\right) \end{array}$$

(3) $$\begin{array}{r} \psi_{j,k}\left(t\right)=2^{\frac{j}{2}}g\left(2^{j}n-k\right) \end{array}$$

where *t* = 0, 1, 2, ….., *M* − 1 is sampling time point, *j* = 1, 2, ……., *J* is the level of decomposition, *k* = 0, 1, 2, ……., 2^*j*^ − 1 is the translation and *J* = 4 (Gonzalez and Woods, 2002). The detail (*D*_*j*_) and approximation (*A*_*j*_) coefficients at the *j*^*th*^ level are computed as follows:

(4) $$\begin{array}{r} D_{j,k}=X{\left(t\right)}^{*}\;\psi_{j,k}\left(t\right) \end{array}$$

(5) $$\begin{array}{r} A_{j,k}=X{\left(t\right)}^{*}\;\phi_{j,k}\left(t\right) \end{array}$$

where *j* = 1, 2, 3, 4 with *J* = 4 and *k* = 0, 1, 2, …., 2^*j*^ − 1.

The energy of *A*_*j*_ and *D*_*j*_ at each decomposition level *j* = 1, 2, 3, 4 is computed as follows:

(6) $$E_{A_{j}}\;=\sum_{i\;=\;1}^{k_{j}}{\left|A_{j,i}\right|}^{2}$$

(7) $$E_{D_{j}}={\sum_{i\;=\;1}^{k_{j}}\left|D_{j,i}\right|}^{2}$$

where *j* = 1, 2, 3, 4 and $k_{j}=2^{j}-1$,

(8) $$\begin{array}{r} E_{total}=E_{A_{J}}+\sum_{j=1}^{J}E_{D_{j}} \end{array}$$

Relative energy of each band is:

(9) $$\begin{array}{r} E_{r}=\frac{E_{j}}{E_{total}} \end{array}$$

where *E*_*j*_ = *E_D_j__*, *j* = 1, 2, 3, 4.

After decomposing EEG signal, we extracted the features using two different approaches:

- Statistical Wavelet Features (SWF),
- Wavelet Coefficients Features (WCF).

#### Statistical wavelet features

After the decomposition (upto level *J* = 4) of each component *s* = {*f*(*t*), *p*(*t*), *c*(*t*)} of *X*(*t*), we obtained approximation coefficients $A_{4}^{s}$ and detail coefficients $D_{4}^{s},\;D_{3}^{s},\;D_{2}^{s}\text{ and }D_{1}^{s},$ the total number of these coefficients was equal to the total number of samples in *s*. From each of $A_{4}^{s},D_{4}^{s},\;D_{3}^{s},\;D_{2}^{s}\text{ and }D_{1}^{s},$ we computed six statistical features [i.e., relative energy (*E*_*r*_), mean (*m*) standard deviation (*std*), kurtosis (*k*), skewness (*sk*) and entropy (*e*)] and formed a feature vector of dimension 90 for three components. When we divided each component upto level 4, we obtained five bands. Then we extracted six features six from each band. It means that there are 30 features for each component of *X*(*t*). As there are three components, *s* = {*f*(*t*), *p*(*t*), *c*(*t*)}, so, there are total number of 90 features for five bands and three components. These features form the feature vector, which represents a trial. These feature vectors were selected from all the trials of the subjects. Therefore, by utilizing all the three components, feature vector is:

(10) $$\begin{array}{r} V=\left[E_{r},\;m,\;std,\;k,\;sk,\;e\right] \end{array}$$

We tested the effect of $A_{4}^{s},D_{4}^{s},\;D_{3}^{s},\;D_{2}^{s}\text{ and }D_{1}^{s}\;$individually for each component *s* = {*f*(*t*), *p*(*t*), *c*(*t*)} of *X*(*t*) and with different combinations to find out which combination gives the best discrimination. Also, we tested different combinations of channels *f*(*t*), *p*(*t*) and *c*(*t*) and different combinations of statistical features.

#### Wavelet coefficients features

In WCF, we used the approximation coefficients $A_{4}^{s}$ and detail coefficients $D_{4}^{s},\;D_{3}^{s},\;D_{2}^{s}\text{ and }D_{1}^{s}$ that were extracted from each component *s* = {*f*(*t*), *p*(*t*), *c*(*t*)} of *X*(*t*). SWF features were computed using statistics of these coefficients. Now the question is whether these coefficients have discriminatory information and can be used directly as features. For this purpose, we used the WT coefficients directly as features.

After the decomposition (upto level *J* = 4), we obtained 153 coefficients from $A_{4}^{s},D_{4}^{s},\;D_{3}^{s},\;D_{2}^{s}\text{ and }D_{1}^{s}\;$for each component *s* = {*f*(*t*), *p*(*t*), *c*(*t*)} of *X*(*t*). To select which coefficients are discriminative, we proposed two techniques:

- With thresholding (WT),
- Without thresholding (WoT).

Intuitively, the small coefficients indicate that the small amount of corresponding frequencies is present in the signal and such frequencies may be unimportant for discrimination. In WT technique, we threshold the extracted coefficients to eliminate the coefficients having values below a threshold E. We tested four different values of threshold, i.e., E= 0.5, 0.1, 0.01, 0.001 to see the effect of thresholding. For thresholding, we used the algorithm presented in Stollnitz et al. (1995).

In WoT technique, we used all coefficients without eliminating the small ones. The purpose was to check whether eliminating the small coefficients affects the prediction accuracy.

### Feature selection

Some SWF features may not be relevant and discriminative. Redundant features not only cause the curse of the dimensionality problem but also affect the prediction accuracy. In this section, we discussed the selection of discriminatory SWF features. The purpose is to select relevant features and decrease the dimension of the feature space by removing redundant features. For features selection, we used Area under ROC curve that is a robust and efficient method for the selection of relevant features. This method selects only the discriminatory features by calculating their importance (Theodoridis and Koutroumbas, 2009).

In addition, we tested experimentally all the possible combinations of extracted features. We also examined different combinations of three channels and sub-bands (*A*_4_ and *D*_1_ to *D*_4_). By using these two methods, we selected two features, i.e., *E*_*r*_ and *std*, which gave the higher prediction accuracy. Table 2 represents *f*(*t*) channel's sub-band percentage *E*_*r*_ and its frequency range of a subject against the single trial.

**Table 2:** **D_1_ to D_4_ represents the detail coefficients from level 1 to 4 and A_4_ represents approximation coefficient at level 4**.

**Levels**	**Wavelet coefficients**	**Frequency bands (Hz)**	**Wavelet relative energy (E**_**r**_**) (%)**	
			**2D**	**3D**
1	D1	15–30	0.64	1.13
2	D2	7.5–15	2.43	4.1
3	D3	3.75–7.5	3.62	9.3
4	D4	1.875–3.75	18.57	8.38
4	A4	0–1.875	74.74	77.09

### Classification

To classify the LA and HA groups is a two-class classification problem. Support Vector Machines (SVM) showed excellent performance in many two-class problems, and as such we used it in our system. It is based on large margin theory and uses the decision function with maximum margin. Though SVM is linear classifier but with kernel trick, it successfully handles non-linearly separable data. Commonly used kernel function is radial basis function (RBF), which gives good performance in a large number of applications, so we used soft SVM with RBF kernel, which has two parameters, i.e., soft margin parameter *C* and RBF kernel parameter (γ). These parameters were tuned using grid search method (Hussain et al., 2011) and five-fold cross-validation.

## Experimental results and discussion

To discuss the results of the proposed scheme, firstly, we give the evaluation protocol, then we discuss the selection of frequency bands using WCF features. After that, results with SWF features have been presented and discussed. Finally, the discrimination of SWF features has been analyzed using statistical significance tests and inter-class and intra-class variations.

### Evaluation protocol

To evaluate the classification system, we used tenfold cross validation technique where the given data was divided into 10-folds. Each fold was held out in turn and remaining 9-folds were used to train and tune the system. After training and tuning the system, the left-over fold was used as an independent set to test the performance of the system. This process was repeated for each fold and average performance values were calculated. The main advantage of this technique was that the system was tested under various samples of data.

To tune the parameters *C* and γ of SVM, we used 25% of the training data, five cross validation and grid search.

For evaluating the performance of the system, we employed the commonly used measures, such as accuracy, precision, sensitivity and specificity, which are defined below:

(11) $$\begin{array}{r} \text{Accuracy}=\frac{\begin{array}{c} \text{Total No}.\text{ of Correctly}\\ \text{Classified Instances} \end{array}}{\text{Total No}.\text{ of Instances}}\times 100 \end{array}$$

(12) $$\begin{array}{r} \text{Precision}=\frac{\text{TP}}{\text{TP}+\text{FP}}\;\times 100 \end{array}$$

(13) $$\begin{array}{r} \text{Sensitivity}=\frac{\text{TP}}{\text{TP}+\text{FN}}\;\times 100 \end{array}$$

(14) $$\begin{array}{r} \text{Specificity}=\frac{\text{TN}}{\text{TN}+\text{FP}}\;\times 100 \end{array}$$

Where,

TP: True Positive: A subject predicted with LA when he/she actually has LA,

TN: True Negative: A subject predicted with HA when he/she actually has HA,

FP: False Positive: A subject predicted with LA when he/she actually has HA, and,

FN: False Negative: A subject predicted with HA when he/she actually has LA.

In 2D case, the total number of trials (epochs) for HA group was of 551, whereas this number was 482 for LA group. Similarly, in 3D case, the total number of trials was 433 for HA group and 529 for LA group.

### Selection of wavelet bands

After decomposition of *X*(*t*), we checked $A_{4}^{s},D_{4}^{s},\;D_{3}^{s},\;D_{2}^{s},\text{ and }D_{1}^{s}$ by plotting different trials of LA and HA group, and observe the maximum discrimination in $A_{4}^{s}\text{ and }D_{4}^{s}$ which represents the delta low and delta high band. On the other hand, we also performed experiments on all decomposition levels with SWF and WCF features, and found that it is in accordance with our above observation that $A_{4}^{s}\text{ and}{\;D}_{4}^{s}$ have discriminative information. We checked all the bands, and best performance is coming from delta band. The prediction accuracy for$\;A_{4}^{s}\text{ and }D_{4}^{s}$, i.e., 100% were higher than the $D_{3}^{s},\;D_{2}^{s}\text{ and }D_{1}^{s}$ components. The accuracy rate for $D_{3}^{s},\;D_{2}^{s}\text{ and }D_{1}^{s}$ was below 83%. From previous studies (Dimitriadis et al., 2010; Harper et al., 2014), the low-frequency delta bands have also been noted by the research scientists as cognitive rhythms, and they associated these bands with attention and cognitive demanded tasks. Based on prediction results and previous studies, we decided to use $A_{4}^{s}\text{ and}{\;D}_{4}^{s}$ (delta low and delta high bands) for proposed system.

### Performance with wavelet coefficients features

To analyze the performance of the classifier, we used WCF features extracted at decomposition level-4. We performed the classification directly on the coefficients, instead of the features, which were obtained after wavelet decomposition at level 4 (*A*_4_ and *D*_4_) by using three channels {*f*(*t*), *p*(*t*), *c*(*t*)} with 2D and 3D contents. The first purpose of this analysis is to check the importance of these coefficients, whether these have discriminatory information or not. The second purpose of this analysis is to compare the accuracy results of WCF with the accuracy rate of the SWF. At DWT decomposition level 4, we obtained 27 coefficients (nine from each channel) from *A*_4_ and *D*_4_ (delta low and delta high sub-bands) separately. Then, we used the SVM classifier on these extracted coefficients by two ways, i.e., (i) with thresholding (WT) and (ii) without thresholding (WoT). The reason to apply a threshold on the extracted coefficients was to eliminate those coefficients that having very small values. In WT technique, we applied four different values of threshold, i.e., E= 0.5, 0.1, 0.01, 0.001. In WoT technique, we applied the SVM classifier directly on the extracted coefficients without eliminating the small ones. Results are shown in Tables 3, 4 for *A*_4_ and *D*_4_ coefficients with 2D and 3D contents separately.

**Table 3:** **SVM Classifier Results (with RBF Kernel) in Classification of HA vs. LA (2D Content) with and without thresholding (for A_4_ and D_4_ coefficients), No. of channels = 03 (*Fz, Pz, Cz*)**.

**Epsilon**	**With thresholding**								**Without thresholding**	
	**0.5**		**0.1**		**0.01**		**0.001**			
	**A_4_**	**D_4_**	**A_4_**	**D_4_**	**A_4_**	**D_4_**	**A_4_**	**D_4_**	**A_4_**	**D_4_**
Accuracy (%)	82	90	88	90	86	84	88	94	97.5	97.9
Sensitivity (%)	79	89	87.2	86	84	83	86	93	98	98.60
Specificity (%)	77.20	86.8	85.1	84.6	82.4	80.2	83.3	92.8	96.8	97.10
Precision (%)	77.60	86.9	85.4	84.9	82.5	80.3	83.7	92.9	96.9	97.4
AUC	0.825	0.93	0.85	0.925	0.88	0.92	0.8	0.95	0.97	0.975
C	8	2.83	8	8	11.3	2	2.83	8	2	2
γ	0.0014	0.004	0.002	0.002	0.0014	0.0055	0.002	0.0055	0.002	0.001

**Table 4:** **SVM Classifier Results (with RBF Kernel) in Classification of HA vs. LA (3D Content) with and without thresholding (for A_4_ and D_4_ coefficients), No. of channels = 03 (*Fz, Pz, Cz*)**.

**Epsilon**	**With thresholding**								**Without thresholding**	
	**0.5**		**0.1**		**0.01**		**0.001**			
	**A_4_**	**D_4_**	**A_4_**	**D_4_**	**A_4_**	**D_4_**	**A_4_**	**D_4_**	**A_4_**	**D_4_**
Accuracy (%)	98	94	92	96	92	98	98	96	98	98.8
Sensitivity (%)	100	93.9	92.6	95.7	91.8	98.6	99	95.8	99.1	99.6
Specificity (%)	96.3	92.5	92.1	94.8	90.3	97.4	98.1	94.7	98.2	98.3
Precision (%)	96.4	92.7	92.4	94.9	90.8	97.5	98.6	94.8	98.3	98.5
AUC	0.975	0.94	0.89	0.97	0.95	0.97	0.97	0.96	0.975	0.98
C	32	2	8	8	8	32	128	4	2	2
γ	0.003	0.0055	0.002	0.0055	0.0055	0.004	0.0007	0.004	0.0014	0.001

**Figure 8:**
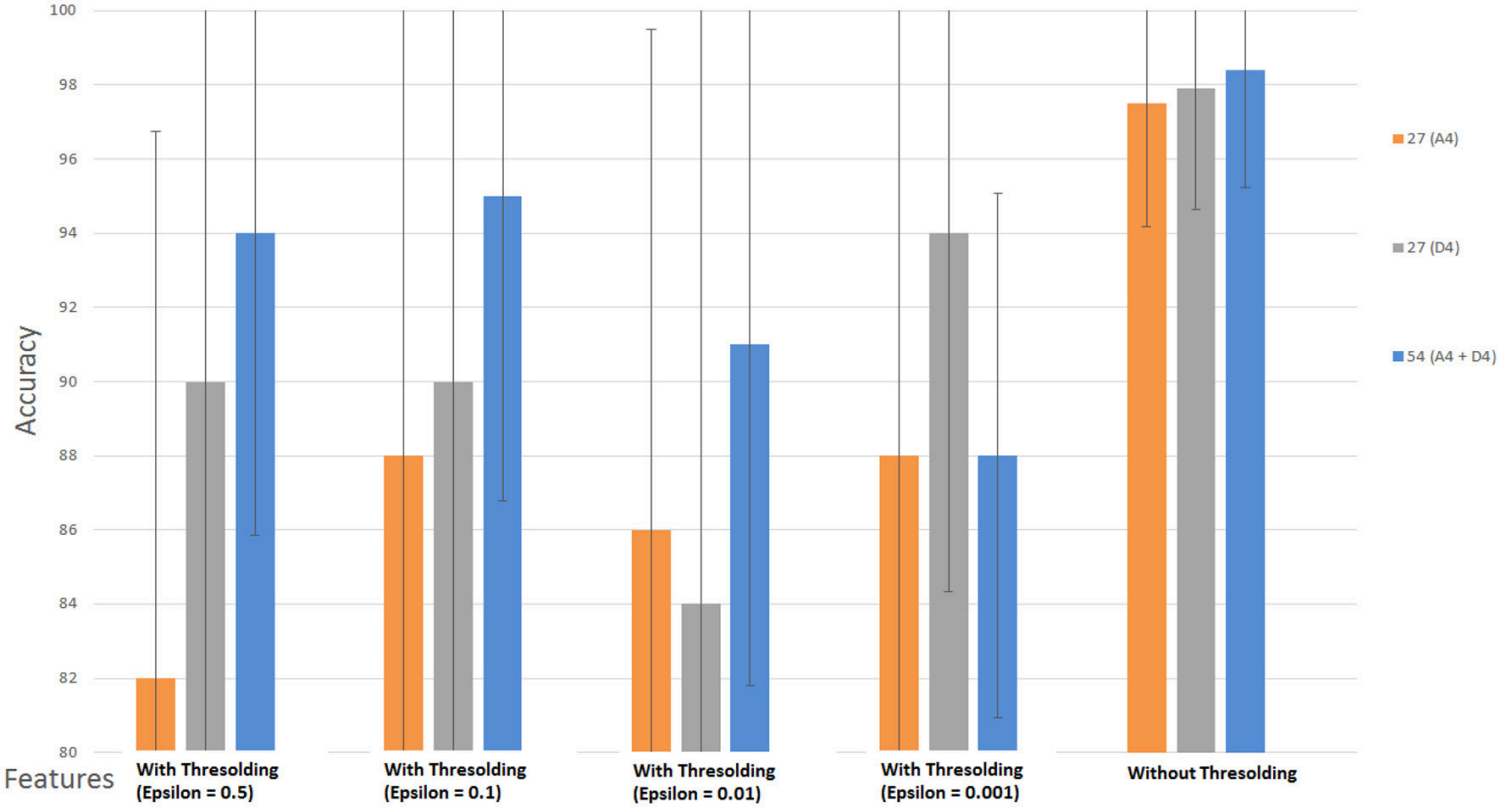
**Classification results of WCF features with and without thresholding using approximate (A4) and detailed coefficients (D4) for the cognitive task with 2D content (HA vs. LA)**. Wavelet type = haar, level = 4, No. of channels = 03 (*Fz, Pz, Cz*).

**Figure 9:**
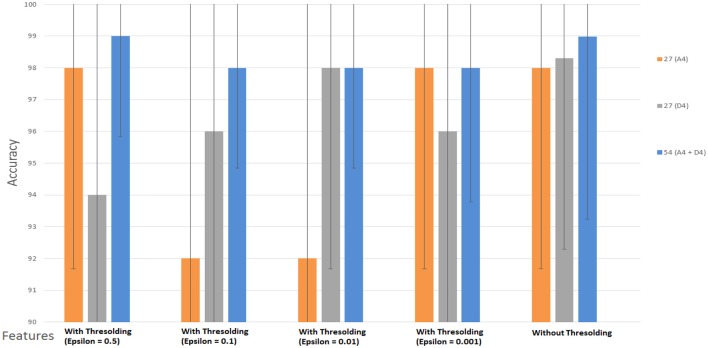
**Classification results of WCF features with and without thresholding using approximate (A4) and detailed coefficients (D4) for the cognitive task with 3D content (HA vs. LA)**. Wavelet type = haar, level = 4, No. of channels = 03 (*Fz, Pz, Cz*).

**Figure 10:**
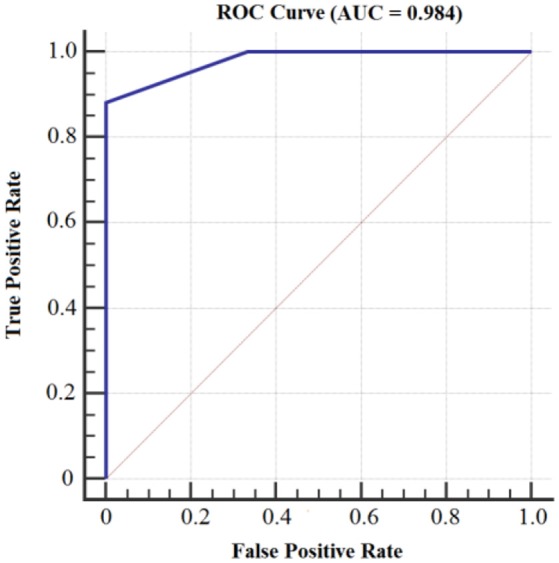
**AUC area under the receiver operating characteristic curve for 2D Content (HA vs. LA) without thresholding**.

**Figure 11:**
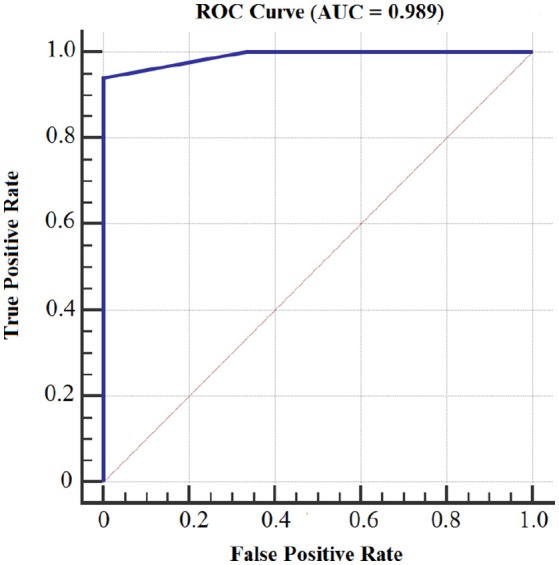
**AUC area under the receiver operating characteristic curve for 3D Content (HA vs. LA) without thresholding**.

Figures 8, 9 show the accuracy of classifier against the coefficients extracted at wavelet decomposition level four for three channels with 2D and 3D contents. The best accuracy achieved by SVM classifier was 98.4 and 98.98% at *A*_4_ and *D*_4_ in 2D and 3D, respectively. Figures 10, 11 show the AUC area under the receiver operating characteristic curve for 2D (HA vs. LA) and 3D (HA vs. LA) without thresholding.

### Performance with statistical wavelet features

To analyze the performance of classifier by using SWF features in various combinations, we used SVM with RBF kernel to classify the LA and HA classes with 2D and 3D contents separately. Our classification technique was used for all the features extracted from all the sub-bands upto level 4, i.e., *A*_4_ and *D*_1_ to *D*_4_. However, the classifier results were not up to the mark for all the sub-bands. The classifier showed the best performance for two out of six extracted features, i.e., *E*_*r*_ and *std*, each from the *A*_4_ and *D*_4_ coefficients at decomposition level 4 for both 2D and 3D contents. *A*_4_ and *D*_4_ represent the delta low and delta high band. The accuracy rate for the other bands, i.e., $D_{3}^{s},\;D_{2}^{s}\text{ and }D_{1}^{s}$ was below 83 %. These results showed the domination of delta-low frequency (0.0–1.875) and delta high frequency (1.875–3.75 Hz) in the cognitive task. Results are shown in Tables 5–8 for *A*_4_ and *D*_4_ coefficients separately. In both 2D and 3D cases, best results were obtained for two features (*E*_*r*_ and *std*) for each of the three channels {*f*(*t*), *p*(*t*), *c*(*t*)} of *X*(*t*) for *A*_4_ and *D*_4_. Therefore, the total number of features was six (06) by using all three channels in 2D and 3D. By using these parameters, we obtained 100 % accuracy in both 2D and 3D scenarios.

**Table 5:** **SVM Classifier Results (with RBF Kernel) in Classification of HA vs. LA (2D Content) with SWF features for A_4_ (delta low), No. of channels = 03 (*Fz, Pz, Cz*)**.

**No. of Features**	**Accuracy (%)**	**Sensitivity (%)**	**Specificity (%)**	**Precision (%)**	**AUC**	**C**	**γ**
*6(Er, std)*	100	100	98.8	98.9	1	8	0.011
*9(Er, std, m)*	90	89	86.2	86.3	0.89	2048	0.00004
*9(k, sk, e)*	80	78.5	77.2	77.5	0.79	512	0.016
*12(Er, k, sk, e)*	90	88	87.7	87.9	0.89	512	0.016
*15(Er, std, k, sk, e)*	86.67	85	84	84.2	0.86	512	0.016
*18(Er, std, m, k, sk, e)*	70	69	68.3	68.9	0.70	2048	0.0055

*Er, Energy; std, Standard Deviation; m, Mean; k, Kurtosis; sk, Skewness; e, Entropy*.

**Table 6:** **SVM Classifier Results (with RBF Kernel) in Classification of HA vs. LA (2D Content) with SWF features for D_4_ (delta high), No. of channels = 03 (*Fz, Pz, Cz*)**.

**No. of Features**	**Accuracy (%)**	**Sensitivity (%)**	**Specificity (%)**	**Precision (%)**	**AUC**	**C**	**γ**
*6(Er, std)*	100	100	98.60	98.80	1.0	2	0.0014
*9(Er, std, m)*	90	91	88.10	88.20	0.88	2	0.0055
*9(k, sk, e)*	80	79	77.40	77.90	0.80	11.3	0.0014
*12(Er, k, sk, e)*	85	82	81.10	81.60	0.84	8192	0.0055
*15(Er, std, k, sk, e)*	80	78	77.30	77.60	0.79	42.3	0.0055
*18(Er, std, m, k, sk, e)*	73.33	71	70.70	70.90	0.73	2048	0.0052

*Er, Energy; std, Standard Deviation; m, Mean; k, Kurtosis; sk, Skewness; e, Entropy*.

**Table 7:** **SVM Classifier Results (with RBF Kernel) in Classification of HA vs. LA (3D Content) with SWF features for A_4_ (delta low), No. of channels = 03 (*Fz, Pz, Cz*)**.

**No. of Features**	**Accuracy (%)**	**Sensitivity (%)**	**Specificity (%)**	**Precision (%)**	**AUC**	**C**	**γ**
*6(Er, std)*	100	100	98.20	98.40	1.0	2	0.0312
*9(Er, std, m)*	90	92	88.40	88.50	0.89	512	0.011
*9(k, sk, e)*	90	89	88.10	88.30	090	512	0.011
*12(Er, k, sk, e)*	90	89.10	88.50	88.60	0.89	512	0.011
*15(Er, std, k, sk, e)*	90	91.40	87.70	87.90	0.91	512	0.011
*18(Er, std, m, k, sk, e)*	76.66	76	75.20	75.50	0.76	512	0.016

*Er, Energy; std, Standard Deviation; m, Mean; k, Kurtosis; sk, Skewness; e, Entropy*.

**Table 8:** **SVM Classifier Results (with RBF Kernel) in Classification of HA vs. LA (3D Content) with SWF features for D_4_ (delta high), No. of channels = 03 (*Fz, Pz, Cz*)**.

**No. of Features**	**Accuracy (%)**	**Sensitivity (%)**	**Specificity (%)**	**Precision (%)**	**AUC**	**C**	**γ**
*6(Er, std)*	100	100	98.50	98.60	1	2	0.0005
*9(Er, std, m)*	90	89.10	88.30	88.60	0.88	512	0.011
*9(k, sk, e)*	90	91.20	88.70	88.80	0.89	8	0.0028
*12(Er, k, sk, e)*	80	80.50	78.10	78.30	0.79	32	0.0055
*15(Er, std, k, sk, e)*	90	89.70	87.60	87.90	0.898	42.3	0.0055
*18(Er, std, m, k, sk, e)*	66.67	66.50	64.20	64.50	0.66	11.3	0.0055

*Er, Energy; std, Standard Deviation; m, Mean; k, Kurtosis; sk, Skewness; e, Entropy*.

**Figure 12:**
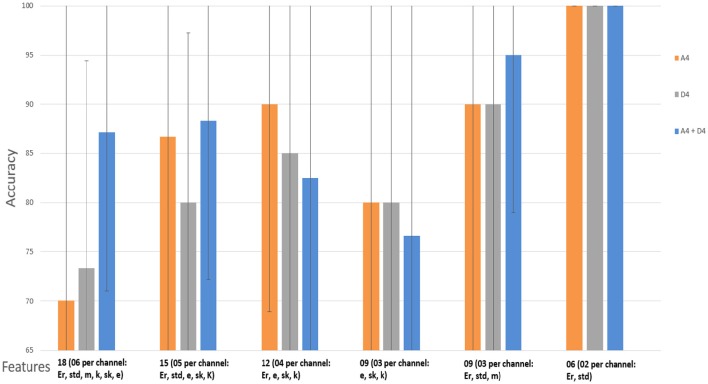
**Classification results of SWF features using approximate (A4) and detailed (D4) coefficients for the cognitive task with 2D content (HA vs. LA)**. Wavelet type = haar, level = 4, No. of channels = 03 (*Fz, Pz, Cz*). Er, Energy; std, Standard Deviation; m, Mean; k, Kurtosis; sk, Skewness; e, Entropy.

**Figure 13:**
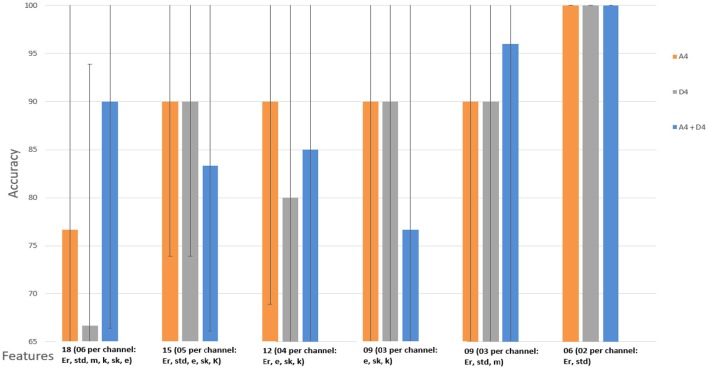
**Classification results of SWF features using approximate (A4) and detailed coefficients (D4) for the cognitive task with 3D content (HA vs. LA)**. Wavelet type = haar, level = 4, No. of channels = 03 (*Fz, Pz, Cz*). Er, Energy; std, Standard Deviation; m, Mean; k, Kurtosis; sk, Skewness; e, Entropy.

**Figure 14:**
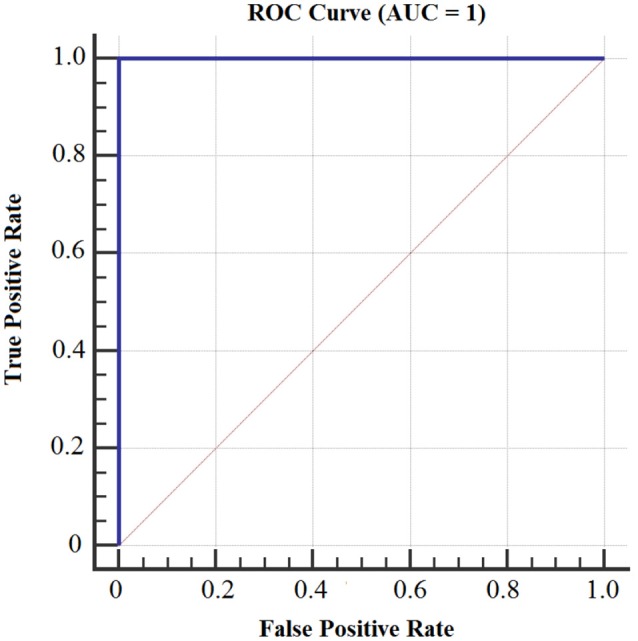
**AUC area under the receiver operating characteristic curve for 2D (HA vs. LA)**.

**Figure 15:**
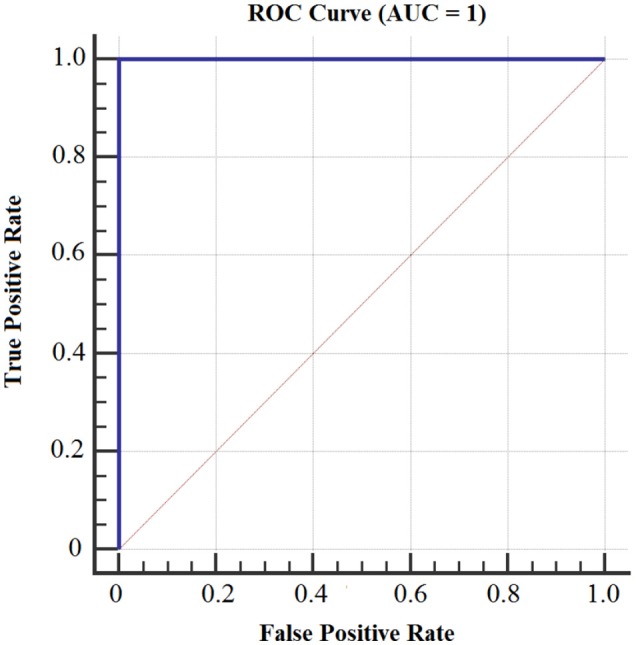
**AUC area under the receiver operating characteristic curve for 3D (HA vs. LA)**.

Figures 12, 13 show the accuracy of classifier against the different number of features for both 2D and 3D cases at wavelet decomposition level 4. The best accuracy achieved by SVM classifier was 100% at *A*_4_ and *D*_4_ by using two features (*E*_*r*_ and *std*) for each channel in both 2D and 3D cases. Figures 14, 15 show the AUC area under the receiver operating characteristic curve for 2D (HA vs. LA) and 3D (HA vs. LA).

### Performance using different subjects for training and testing data

In our evaluation so far, we used all trials together from all subjects and divided it into training and testing data using 10-fold cross validation to test the performance of the system over different variations of the data. To show that the performance of the system does not depend on subjects, we also performed experiments by using testing data from the subjects, from whom the data was not used for training i.e., we used training and testing data from different subjects. For this purpose, we used the data from 70% of the subjects as training and the data from the remaining 30% of the subjects as testing, for both 2D and 3D cases. Using the features found to be the best in previous sections, i.e., *E*_*r*_ and *std* of *A*_4_ and *D*_4_ sub-bands, the accuracy achieved by SVM classifier is 100% for both 2D and 3D cases. It indicated that the performance of the system is independent of subjects.

### Analysis of discrimination of features

To analyze the discrimination of features giving the best performance, we used the following two different approaches:

1. Statistical significance test,
2. Inter-class and Intra-class variation analysis.

#### Statistical significance test

We used the statistical test to check the significance of our discriminatory SWF features. For this purpose, we performed the non-parametric procedure, i.e., Kruskal-Wallis Anova test. This test was basically used to compare the means between three or more independent groups. We applied this test in both 2D and 3D cases to check whether there is a significant difference between the features of LA and HA groups or not. For this purpose, we formed a hypothesis in 2D case, i.e.,

***Null Hypothesis H***_0_: No significant difference exists between HA and LA classes in 2D.
***Alternate Hypothesis H***_1_: Significant difference exists between HA and LA classes in 2D.

**Figure 16:**
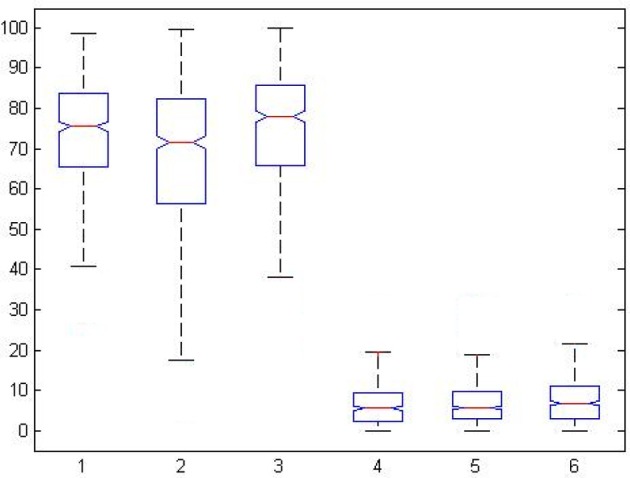
**Kruskal-Wallis Anova test for 2D case (HA vs. LA)**.

We took three samples each from HA (sample # 1 to 3) and LA classes (sample # 4 to 6) from *E*_*r*_ and *std* features, and applied Kruskal-Wallis test on these samples. Figure 16 shows the result of Kruskal-Wallis test.

After performing the test, we got the *p* = 0 which was less than alpha (0.05), therefore, we rejected the null hypothesis, and we can say that significant difference exists between HA and LA classes in 2D case. From Figure 16, we can also observe that first three samples are grouped together belonging to HA class (2D) and last three samples are grouped together belonging to LA class (2D). The samples of same class are close to each other; however, there is significant difference between the samples of different classes. Therefore, alternate hypothesis exists.

In 3D case, we also formed a hypothesis.

***Null Hypothesis H***_0_***:*** No significant difference exists between HA and LA classes in 3D.
***Alternate Hypothesis H***_1_***:*** Significant difference exists between HA and LA classes in 3D.

**Figure 17:**
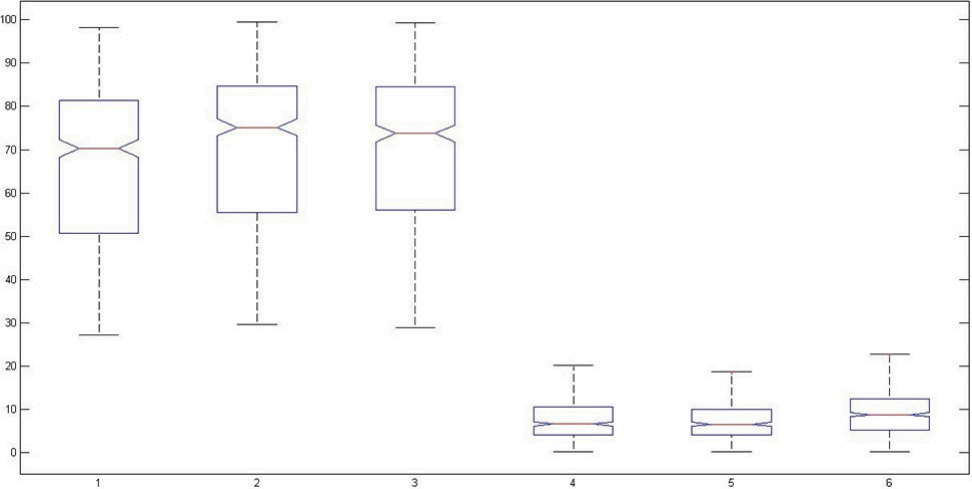
**Kruskal-Wallis Anova test for 3D case (HA vs. LA)**.

Similarly, we took three samples each from the HA (sample # 1 to 3) and LA classes (sample # 4 to 6) from *E*_*r*_ and *std* features and applied Kruskal-Wallis test on these samples. Figure 17 shows result of Kruskal-Wallis test.

After performing the test, we obtained the *p* = 2.3 × 10^−9^ which was less than alpha (0.05) so we rejected the null hypothesis, and we can say that significant difference exists between HA and LA classes in 3D case. From Figure 17, we can observe that first three samples are grouped together belonging to HA class (3D) and last three samples are grouped together belonging to LA class (3D). The samples of same class are close together; however, there is significant difference between the samples of different classes. Therefore, alternate hypothesis exists.

#### Inter-class and intra-class variation analysis

We used the inter-class and intra-class variation analysis to show the significant difference between the SWF of LA and HA groups. For this analysis, we used the scatter matrices (Theodoridis and Koutroumbas, 2006) in 2D case. We calculated within-class scatter matrix (*S*_*w*_) and between-class scatter matrix (*S*_*b*_) of LA and HA groups by taking the samples from the *E*_*r*_ and *std* features. After getting the *S*_*w*_ and *S*_*b*_, we calculated the mixture scatter matrix (*S*_*m*_), i.e.,

(15) $$\begin{array}{r} S_{m}=S_{w}+S_{b} \end{array}$$

Where *S*_*m*_ represents the covariance matrix of the feature vector according to global mean. Then, we calculated the trace and determinant of *S*_*m*_ and *S*_*w*_. The criteria to check the significant difference between the features of two classes is that the trace {*S*_*m*_} should be greater than trace {*S*_*w*_}, i.e., *J*_1_, and determinant {*S*_*m*_} should also be greater than determinant {*S*_*w*_}, i.e., *J*_2_. It is represented as:

(16) $$\begin{array}{r} J_{1}=\frac{trace\;\left\{S_{m}\right\}}{trace\;\left\{S_{w}\right\}} \end{array}$$

(17) $$\begin{array}{r} J_{2}=\frac{det\;\left\{S_{m}\right\}}{det\;\left\{S_{w}\right\}} \end{array}$$

It took larger values when samples in the L–dimensional space are well clustered around their mean, within each class and the clusters of the different classes are well separated. After using the Equations 16 and 17, we got *J*_1_ = 11.0508 and *J*_2_ = 2.2570 × 10^3^. This shows that criteria which define the condition, i.e., trace {*S*_*m*_} > trace {*S*_*w*_} and det {*S*_*m*_} > det {*S*_*w*_} holds. Therefore, analytical analysis shows that significant difference exists between the features of LA and HA groups in 2D case.

### Analysis of EEG signals

**Figure 18:**
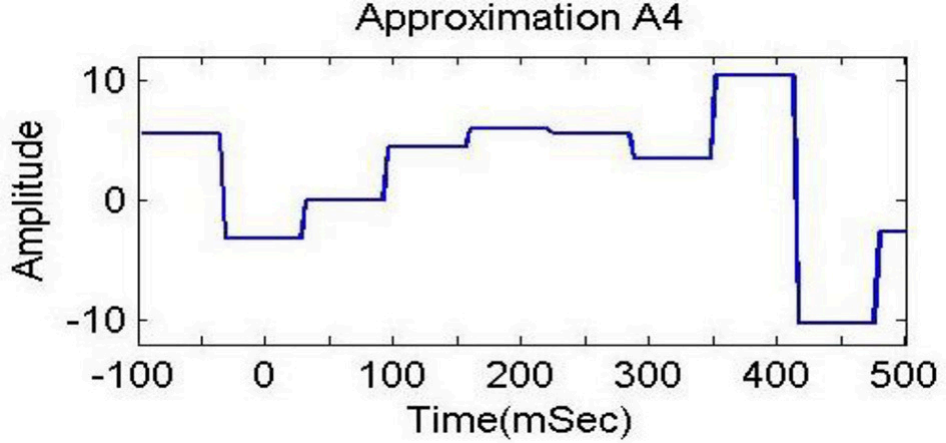
**Delta low (A_4_) in 0.0 to 1.875 Hz in HA (2D content)**.

**Figure 19:**
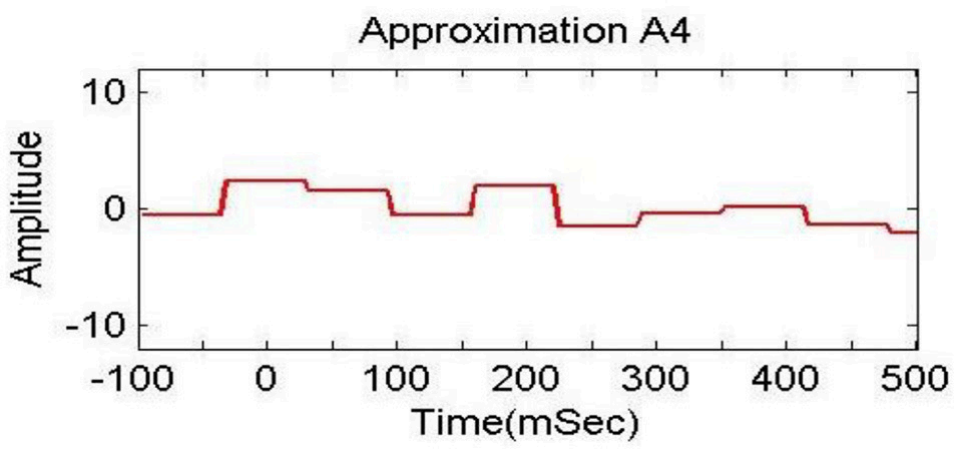
**Delta low (A_4_) in 0.0 to 1.875 Hz in LA (2D content)**.

**Figure 20:**
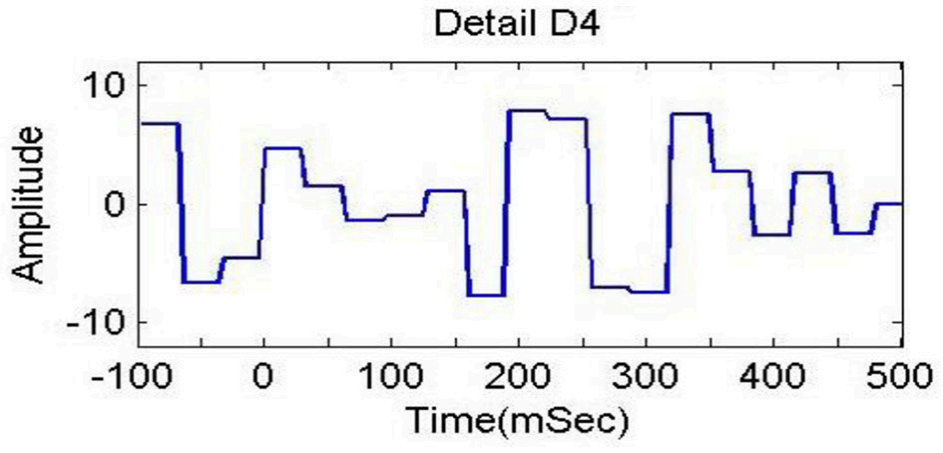
**Delta high (D_4_) in 1.875 to 3.75 Hz in HA (2D content)**.

**Figure 21:**
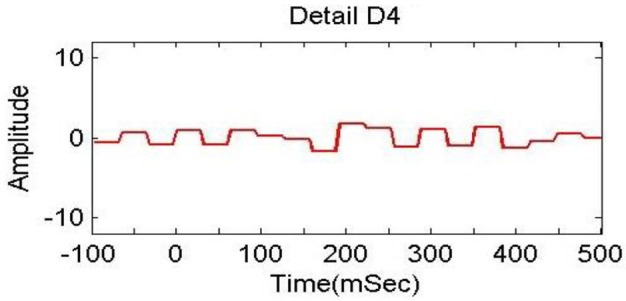
**Delta high (D_4_) in 1.875 to 3.75 Hz in LA (2D content)**.

To analyze the discrimination of EEG signals of LA and HA classes, we used different combinations of features in classification of SWF features, such as 6, 9, 12, 15, and 18 for our classification problem by utilizing all the three channels and both *A*_4_ and *D*_4_ coefficients at level 4 by considering 2D and 3D contents. We used different performance measuring parameters to check the result of our pattern recognition system like accuracy, sensitivity, specificity and precision. From the Tables 5–8, we can observe that SVM classifier got the 100% accuracy rate in both 2D and 3D cases. Experimentally, we observed the classification rate by taking different number of features as mentioned in Figures 12, 13. We got the best classification rates, i.e., 100% for both 2D and 3D cases by using the two features, i.e., *E*_*r*_ and *std*. These results show the discrimination between LA and HA classes by performing the cognitive task using the 2D and 3D content. A signal obtained from *Fz* channel by performing the cognitive task for the duration of 600 mS in 2D case is shown in Figures 18–21. From the figures, we can analyze the clear difference between the amplitude of the signals in the delta low (0.0–1.875) and delta high (1.875–3.75) frequency bands for LA and HA classes in 2D case. These differences of amplitude verify that these frequency bands, i.e., delta low and delta high have discriminatory features (*E*_*r*_ and *std*) which help us to classify the LA and HA classes in 2D and 3D cases.

In classification of WCF features with and without thresholding, the best accuracy rate achieved by SVM classifier was 98.4 and 98.98% at A_4_ and D_4_ in 2D and 3D, respectively, as shown in Figures 8, 9. However, by applying the thresholding in 2D case, we can see in Figure 8 that its accuracy rate has been affected; however, without thresholding; its accuracy rate was much higher. It means that discriminatory information also exists in the small values of the coefficients. Therefore, all the coefficients extracted from the sub-bands at level-4 may be used for feature extraction in 2D case. In 3D case, we can see that from Figure 9, the accuracy rates have not been affected with and without thresholding. It gives almost the same accuracy in both cases. It means that elimination of coefficients in 3D case does not affect the accuracy rate. Therefore, we can use the coefficients obtained after the thresholding for the extraction of features.

In this study, we used three midline channels for the recording of EEG signals, i.e., *Fz, Pz, Cz*. After getting the classification results as shown in Figures 12, 13, we can observe that these three channels play an important role in recording of signals during the cognitive task. From these signals, we extracted the discriminatory features to use in the classification problem. After achieving these results, we can conclude that the features, i.e., *E*_*r*_ and *std*, which are extracted from the delta low (0.0 to 1.875 Hz) and delta high (1.875 to 3.75 Hz) frequency bands, can be used for the classification of EEG brain patterns recorded during the cognitive task.

### Analysis of frequency bands

In this study, we extracted the two discriminatory features (*E*_*r*_ and *std*) from delta low and delta high bands, which were obviously low frequency bands, and then these features were used during the classification process. From 100% results that we achieved in this research study, we observed the importance of EEG low frequency bands in the classification problem. In Dimitriadis et al. (2010), Amin and Malik (2013), and Harper et al. (2014), the low-frequency EEG bands have also been noted by the research scientists as cognitive rhythms, and they associated these bands with attention and cognitive demanded tasks.

In ERP research studies (Ergen et al., 2008; Harper et al., 2014), researchers highlighted the importance of delta band by associating the P300 component with cognitive processes. In the neuroscience research literature, this association was extensively discussed. Delta band has also very important contribution toward the ERP's P300 Component (Demiralp et al., 1999). In another study (Gennady, 2012), authors confirmed the association between the delta band and cognitive processing. Some of the above-discussed studies showed the increase in the delta power during the cognitive tasks. Our research study also validates the findings of Amin et al. (2015a) to assess an individual's learning and memory recall ability based on P300 component. Due to above-mentioned reasons, we got the best accuracy rates during the classification process of EEG low frequency bands, i.e., delta low (0.0–1.875 Hz) and delta high (1.875–3.75 Hz) for discriminating the HA and LA ability groups in 2D and 3D cases by performing the visual oddball cognitive task.

### Effect of 2D and 3D contents on high ability and low ability groups

From the prediction accuracy, i.e., 100%, we observed that there was no significant difference between the effect of 2D and 3D contents on HA and LA groups. By presenting both the contents, i.e., 2D and 3D to HA and LA groups, we achieved the same accuracy rate, i.e., 100%. However, in order to evaluate the effect of 2D and 3D contents on HA and LA groups statistically, a hypothesis has been developed that measures the system performance related to 2D content whether it is less effective than 3D or not. For this purpose, we formed a hypothesis, i.e.,

***Null Hypothesis H***_0_: No significant difference in the system performance related to 2D and 3D content.
***Alternate Hypothesis H***_1_: System performance related to 2D content is less effective than 3D.

In order to analyze the significant difference between 2D and 3D contents, we needed a large number of samples for accuracy values. Therefore, to meet this requirement, we run our system three times for 10 cross validation by utilizing the best parameters. Each time, we randomized our dataset. This process provides 30 prediction values for each system. In the next step, we utilized the independent t-test. After performing the *t*-test, we got *p* = 1; which indicated that there is no significant difference exists in the system performance related to 2D and 3D contents. Null hypothesis was accepted as we got *p*-value greater than 0.05. Therefore, we conclude statistically that there is no significant difference between the effect of 2D and 3D contents on HA and LA groups.

### Comparisons

To evaluate the effectiveness of our proposed system, we compared our experimental results with previous EEG research studies. However, result comparisons with previous studies are complex due to various decomposition levels, wavelet types, variability of participants, and the variety of cognitive tasks. A detailed comparison of proposed methodology with previous research studies considering the performance of classifier, ML (machine learning) algorithm, cognitive tasks, dataset, and feature extraction methods is presented in Table 9. The research studies mentioned in Table 9 used the frequency domain, time domain, wavelet transform and autoregressive coefficients (AR) based features for the classification of EEG signals recorded during the cognitive task. Some of the research studies, mentioned in the comparison table, used the non-linear classifiers, are complex and more time-consuming to construct the classifier model. In research study (Subasi and Gursoy, 2010), authors used few instances only for the classification that results to create the overfitting issue. In this proposed methodology, we used a large number of trials or samples for each HA and LA classes, i.e., 551 for HA group (2D), 482 for LA group (2D), 433 for HA group (3D), and 529 for LA group (3D). We used a total number of 1995 trials. For each trial, we used six features (two from each channel) for the classification. We also used 10-fold cross validation in the classification process. The advantage of 10-fold cross validation scheme was that all the samples are used in the training and testing phase (Subasi and Gursoy, 2010). Therefore, by using a large number of trials or samples, we can compare our results with the previous studies to check the performance of classifier. From Table 9, we can observe that the results of our study are better than the previous studies, which are using the same or different classifier with the similar nature of the cognitive task.

**Table 9:** **Comparison with existing techniques for EEG in cognitive tasks**.

**Sr. no**.	**References**	**Subjects**	**Scalp electrodes**	**Feature**	**Classifier**	**Accuracy**	**Cognitive task**
1	Our Work	34 (each for 2D and 3D case separately)	128	Relative Energy and standard deviation	SVM with RBF Kernel	100 % (for both 2D and 3D)	RAPM and Visual oddball cognitive task
2	Liang et al., 2006	07	6	Autoregressive coefficients	ELM, SVM and ANN	53.98–56.07	05 tasks as mentioned in Keirn and Aunon (1990)
3	Diez et al., 2009	07	6	Frequency and time domain features	LDA and ANN	87.35–91.17	05 tasks as mentioned in Keirn and Aunon (1990)
4	Lin and Hsieh, 2009	03	8	Power feature	SVM and ANN	65.90–68.35	03 cognitive tasks (words generation, imagination of left and right hand movement)
5	Zhiwei and Minfen, 2007	02	06	Wavelet packet entropy	SVM	87.5–93.0	05 tasks as mentioned in Keirn and Aunon (1990)
6	Daud and Yunus, 2004	04	06	Discrete wavelet transform	ANN	74.40–82.30	05 tasks as mentioned in Keirn and Aunon (1990)
7	Hosni et al., 2007	07	06	Autoregressive coefficients and power of frequency bands	SVM	70	Three tasks (multiplication, baseline and mental letter)
8	Yazdani et al., 2009	04	06	db4 wavelet	K-NN	81.48–89.58	05 tasks as mentioned in Keirn and Aunon (1990)
9	Guo et al., 2011	07	06	Immune feature	SVM	85.40–97.5	05 tasks as mentioned in Keirn and Aunon (1990)
10	Xue et al., 2003	04	06	Wavelet packet	RBF network	85.30	05 tasks as mentioned in Keirn and Aunon (1990)

### Limitations of the study

There are some limitations in the existing study, which will be considered in future research. In this study, all the individuals were male subjects. However, in future study, we will also consider female subjects in our experiment to predict the fluid intelligence level of both the genders. In future study, we will also consider some subjects that might have some medical problems, to analyze the effects of diseases on fluid intelligence level. There were only 34 subjects in the current study; therefore, number of subjects should be increased to prove that single trial EEG signals would be enough to predict the cognitive performance. Also, Raven's Advanced Progressive Matrices (RAPM) norm for university students of different nationalities will also be included in the future study. In addition, this research examined the association of EEG signals with memory and learning ability for young subjects only. In addition, the learning contents utilized in this research were associated to physiology and anatomy material; therefore, the results cannot be generalized to relate with learning capability of many other types of memory recall capability or academic learning materials. Finally, 128-chnanels, a very high-density EEG electrode system utilized in this research study cannot be considered appropriate for recording a few electrode EEG channels. In future study, we will utilize EEG recording devices with fewer electrodes according to the requirements of research.

## Conclusion

In this research paper, we have proposed a system for predicting the fluid intelligence level of subjects, whether he/she belongs to LA and HA group using EEG single trial signals. For this purpose, we employed 2D and 3D contents and 34 subjects each for 2D and 3D, which were divided into low-ability (LA) and high-ability (HA) groups using RAPM test. Then, we used the visual oddball cognitive task to measure the neural activity of each group as three EEG signals (*Fz, Pz, Cz*). In order to predict whether a subject belongs to LA or HA group, we extracted two different types of Haar wavelet transform based features using wavelet decomposition from EEG signals in the band 0.3 to 30 Hz. Then support vector machine (SVM) was used as a classifier with RBF kernel to classify the LA and HA groups in 2D and 3D cases. We achieved the 100% classification rate in 2D and 3D cases with the domination of delta low (0.0–1.875 Hz) and delta high (1.875–3.75 Hz) frequency bands. After getting the best results, we conclude that *E*_*r*_ and *std* are useful features in the classification of EEG brain patterns. In addition, the low-range frequency bands, i.e., delta band, show close association with the cognitive processes. The analysis of these frequency bands indicate clear difference between LA and HA groups. Furthermore, discriminative values of the features have been validated using statistical significance tests and inter-class and intra-class variation analysis. Also, statistical test shows that there is no effect of 2D and 3D content on fluid intelligence level. Comparison with state-of-the-art techniques shows the significance of the proposed system. Therefore, our proposed system will be helpful in academic and clinical applications to predict the intelligence level of the subjects, and it may be implemented in online as well as offline applications.

## Author contributions

EQ and MH designed the study. EQ and HUA designed the experiment and collected the data. EQ developed the methodology. EQ, MH, HAS, AM, and SB performed the statistical analysis. EQ and MH performed the results interpretation and drafted the manuscript. All authors read and approved the final manuscript.

### Conflict of interest statement

The authors declare that the research was conducted in the absence of any commercial or financial relationships that could be construed as a potential conflict of interest.
